# A commentary on ‘Management of traumatic brain injury in Africa: challenges and opportunities’ (Int J Surg 2024; Apr 4: Online ahead of print)

**DOI:** 10.1097/JS9.0000000000001639

**Published:** 2024-05-15

**Authors:** Qiushun Zhang, Yongyi Zhang, Junchen Zhang

**Affiliations:** a School of Clinical Medicine, Jining Medical University; b Department of Neurosurgery, Affiliated Hospital of Jining Medical University, Jining; c School of Pharmacy, Shandong Second Medical University, Weifang, Shandong, People’s Republic of China

*Dear Editor,*

Recently, we read the article ‘Management of traumatic brain injury in Africa: challenges and opportunities’ by Professor Muili *et al*.^1^. Traumatic brain injury (TBI) is a major public health concern globally. The study highlights several key challenges faced by Sub-Saharan Africa (SSA) healthcare systems in managing TBIs, including limited resources, inadequate infrastructure, and a shortage of trained healthcare professionals. Furthermore, social and cultural factors, such as ignorance of driving laws, financial constraints, and limited access to modern technology services. However, the study also identifies potential opportunities for improving TBI management in SSA. These include strengthening healthcare infrastructure, enhancing pre-hospital care and transportation systems, and increasing public awareness and education about TBI.

This perspective study offers a thorough examination of the current landscape of TBI management in SSA, highlighting the unique challenges and potential opportunities. It is a valuable resource for neurosurgeons and governmental bodies globally. The authors are particularly laudable for emphasizing the shortage of specialized neurosurgeons and nurses. They note that in SSA, the TBI mortality rate ranges from 22.6 to 30.7% among all injured patients. From China’s experience, emergency surgery performed by specialized neurosurgeons plays a decisive role in the reduction of craniocerebral injury mortality. In the EU CENTER-TBI project, which involved China, the mortality rate for 11 770 cases of acute traumatic brain injuries in 39 hospitals was 7.31% in level II hospitals without specialized neurosurgeons and 2.75% in level III hospitals with specialized neurosurgeons^2^. We are very grateful for the author’s contribution, but there are still some problems in this study that need to be further explored.

This study focuses solely on the epidemiologic characteristics of TBI in adults. Coulter *et al*.^3^ highlighted the distinct differences in epidemiologic characteristics, diagnosis, and treatment of TBI in children compared to adults. Therefore, it is recommended that future studies consider children as a special group requiring specific attention.

The authors have identified the lack of rehabilitation services as a significant challenge for low- and middle-income countries but have not provided a solution. Functional brain rehabilitation has been shown to effectively enhance the quality of life and decrease disability in patients with craniocerebral injuries^4^. To address this issue, increasing the availability of rehabilitation facilities and providing training for rehabilitation professionals is crucial.

Virk *et al*.^5^ share Sierra Leone’s success in utilizing appropriate financing mechanisms to address patients’ high out-of-pocket expenses in one study. The authors suggest that exploring measures like commercial insurance could potentially benefit individuals in SSA regions, highlighting the need for further research in this area.

Despite the mentioned challenges, we appreciate the authors for their significant contribution to the field, particularly in the management of TBI in Africa. We trust that our recommendations will assist the authors in their future research endeavors and eagerly await their response.

## Ethical approval

Not applicable.

## Consent

Not applicable.

## Sources of funding

This research did not receive any specific grant from funding agencies in the public, commercial, or not-for-profit sectors.

## Author contribution

Q.Z.: wrote the paper; Y.Z.: study design; J.Z.: data analysis.

## Conflicts of interest disclosure

There are no conflicts of interest.

## Research registration unique identifying number (UIN)

Not applicable.

## Guarantor

Junchen Zhang.

## Data availability statement

The correspondence is based exclusively on resources that are publicly available on the internet and duly cited in the ‘References’ section. No primary data were generated and reported in this manuscript. Therefore, the data have not become available to any academic repository.

## Provenance and peer review

Not commissioned, externally peer-reviewed.
